# Evaluation of the Pharmacokinetic Drug–Drug Interaction between Micronized Fenofibrate and Pitavastatin in Healthy Volunteers

**DOI:** 10.3390/pharmaceutics12090869

**Published:** 2020-09-12

**Authors:** Hae Won Lee, Woo Youl Kang, Wookjae Jung, Mi-Ri Gwon, Kyunghee Cho, Dong Heon Yang, Young-Ran Yoon, Sook Jin Seong

**Affiliations:** 1 Department of Molecular Medicine, School of Medicine, Kyungpook National University and Department of Clinical Pharmacology, Kyungpook National University Hospital, Daegu 41566, Korea; haewonbbc@gmail.com (H.W.L.); youwoo2@nate.com (W.Y.K.); wookjaejung@nate.com (W.J.); miri.gwon@gmail.com (M.-R.G.); 2 Analytical Research Division, Biocore Co. Ltd., Seoul 08511, Korea; khcho@bio-core.com; 3 Division of Cardiology, Department of Internal Medicine, School of Medicine, Kyungpook National University, Daegu 41944, Korea; ddhyang@knu.ac.kr

**Keywords:** micronized fenofibrate, pharmacokinetics, drug-drug interaction, pitavastatin, safety

## Abstract

Dyslipidemia is a major risk factor for development of atherosclerosis and cardiovascular disease (CVD). Effective lipid-lowering therapies has led to CVD risk reduction. This study evaluated the possible pharmacokinetic interactions between fenofibrate, a peroxisome proliferators-activated receptors α agonist, and pitavastatin, a 3-hydoxy-3-methylglutaryl-coenzyme A reductase inhibitor, in healthy Korean subjects. The study design was an open-label, randomized, multiple-dose, three-period, and six-sequence crossover study with a 10-day washout in 24 healthy volunteers. It had three treatments: 160 mg of micronized fenofibrate once daily for 5 days; 2 mg of pitavastatin once daily for 5 days; and 160 mg of micronized fenofibrate with 2 mg of pitavastatin for 5 days. Serial blood samples were collected at scheduled intervals for up to 48 h after the last dose in each period to determine the steady-state pharmacokinetics of both drugs. Plasma concentrations of fenofibric acid and pitavastatin were measured using a validated high-performance liquid chromatography with the tandem mass spectrometry method. A total of 24 subjects completed the study. Pitavastatin, when co-administered with micronized fenofibrate, had no effect on the C_max,ss_ and AUC_τ,ss_ of fenofibric acid. The C_max,ss_ and AUC_τ,ss_ of pitavastatin were increased by 36% and 12%, respectively, when co-administered with fenofibrate. Combined treatment with pitavastatin and micronized fenofibrate was generally well tolerated without serious adverse events. Our results demonstrated no clinically significant pharmacokinetic interactions between micronized fenofibrate and pitavastatin when 160 mg of micronized fenofibrate and 2 mg of pitavastatin are co-administered. The treatments were well tolerated during the study, with no serious adverse events.

## 1. Introduction 

Cardiovascular disease (CVD) is one of the major causes of mortality and morbidity worldwide [1]. Dyslipidemia is a critical cause for the initiation and progression of atherosclerosis, a major underlying cause of CVD [2]. Atherosclerotic cardiovascular disease (ASCVD) event risk is associated with the levels of atherogenic cholesterol, non-high-density lipoprotein cholesterol (non-HDL-C) and low-density lipoprotein cholesterol (LDL-C) [3]. However, for patients at low or moderate risk for ASCVD, a trial of lifestyle modification therapies should be attempted prior to the initiation of drug therapy. Alternatively, drug therapy may be started concomitantly with lifestyle-based therapies in patients at high or very high risk of developing ASCVD [3].

According to the cholesterol treatment guidelines to reduce ASCVD risk, statins are used as a first-line therapy in patients with elevated atherogenic cholesterol levels [4,5]. These agents, by competitive inhibition of 3-hydroxy-3-methylglutaryl-coenzyme A reductase, decrease cholesterol synthesis in the liver, increase hepatic LDL-C receptor expression, and increase the uptake of circulating LDL from the blood, leading to a decrease in LDL-C and total cholesterol levels [6]. Statins decrease the risk for ASCVD events by 25–45% through the reduction of LDL-C by 25–55% [4].

Pitavastatin is indicated for the reduction of elevated total cholesterol and LDL-C levels as an adjunctive therapy to diet modification, in patients with primary hypercholesterolemia and mixed dyslipidemia. In several double-blind, randomized phase III studies in patients with primary hyperlipidemia or mixed dyslipidemia, pitavastatin at doses of 1–4 mg, demonstrated noninferior efficacy to simvastatin (10–20 mg), and atorvastatin (10–20 mg) in terms of the improvement of LDL-C levels, and resulted in significantly greater LDL-C reduction in elderly patients compared with pravastatin (10–40 mg) [7,8].

Pitavastatin is rapidly absorbed following oral administration and it achieves peak plasma concentrations (C_max_) in about 1h. It has a relatively long elimination half-life (t_1/2_) of approximately 12 h [7]. Most of the circulating pitavastatin moiety is in the parent nonmetabolized form, and it undergoes metabolism to generate an inactive lactone primarily by uridine 5′-diphosphate (UDP) glucuronosyltransferases (UGT1A3 and UGT2B7) [6,8]. After oral administration, pitavastatin is minimally metabolized by the cytochrome P450 (CYP) 2C9 and to a lesser extent by CYP2C8 [8]. The majority (79%) of administered pitavastatin is excreted through the bile into the feces, with some enterohepatic recirculation, and less than 5% of the dose is excreted in the urine [8,9]. The transporters involved in the hepatic uptake of pitavastatin are organic anion-transporting polypeptide (OATP)1B1 (90% of the total hepatic uptake), OATP2B1, and OATP1B3 [9]. Breast cancer resistance protein (BCRP), P-glycoprotein (P-gp) and multidrug resistance-associated protein (MRP2) play a major role in the intestinal efflux and biliary excretion of pitavastatin [9,10,11].

Fibrates, which act as peroxisome proliferators-activated receptors (PPARs) α agonists, are used therapeutically to decrease the levels of plasma triglycerides, LDL-C, and to increase the levels of HDL-C [12]. In previous randomized controlled clinical trials, fenofibrate monotherapy has been shown to increase HDL-C by about 10–50%, and reduce triglycerides by about 20–50% [13]. Fenofibrate, a pharmacologically inactive pro-drug, is rapidly metabolized to the active metabolite fenofibric acid following oral administration. Subsequently, fenofibric acid is metabolized through conjugation with glucuronic acid [14,15]. For micronized fenofibrate, which was specifically developed to enhance the low bioavailability of fenofibrate, the C_max_ is achieved at 6–8 h after oral administration, with a t_1/2_ of approximately 20 h [14,16]. After absorption, fenofibrate is mostly excreted in the urine in the form of fenofibric acid and fenofibric acid glucuronide [15]. According to several in vitro studies conducted using human liver microsomes, fenofibric acid is a weak inhibitor of CYP2C8, CYP2C19, and CYP2A6, and a mild-to-moderate inhibitor of CYP2C9 when administered therapeutic concentrations [13].

In those patients in whom statins are not tolerated or statin monotherapy is not adequate due to resistance, alternative lipid-lowering therapeutic strategies are needed to lower the residual CVD risk [11]. In patients with mixed dyslipidemia with high triglyceride and low HDL-C levels, combining fibrate with statin was found to be effective in lowering CVD risk by approximately 27% [17]. According to the 2016 European guidelines for the management of dyslipidemias, the addition of a fenofibrate to a statin may be considered in high-risk patients with triglyceride level of more than 200 mg/dL despite being treated with statins [18]. Several studies have demonstrated the beneficial effects of pitavastatin on lipids in patients with type 2 diabetes mellitus and metabolic syndrome with no deleterious effects on glycemic control, on the size and composition of atherosclerotic plaques, improvement in cardiovascular function, and improvements in markers of inflammation, oxidative stress, and renal function [19]. Accordingly, there is a need to evaluate the potential pharmacokinetic drug–drug interactions between pitavastatin and fenofibrate because of the potential benefit of this combination therapy as an alternative to statins monotherapy in this patient population. Based on a literature review (PubMed key terms: pitavastatin, fenofibrate, drug-drug interaction (June 2020)), no research reports on the drug-drug interaction between pitavastatin and fenofibrate have been identified. Therefore, the main objective of this study was to evaluate the potential interactions between pitavastatin and micronized fenofibrate after co-administration and determine the safety and tolerability of this combination.

## 2. Methods

The Institutional Review Board of Kyungpook National University Hospital (KNUH, Daegu, Korea) reviewed and approved the study protocol. The study (Clinical trial registry identifier: NCT01767610) was conducted in accordance with the principles outlined in the Declaration of Helsinki and the Good Clinical Practice guidelines. All subjects were provided with written informed consent to participate in this study before initiating the study.

### 2.1. Study Design and Subjects 

The study population consisted of healthy male volunteers, 20–55 years of age. They were required to have a body mass index of 18 to 29 kg/m^2^, and a minimum body weight of 50 kg. Exclusion criteria included a history of, or ongoing clinically significant medical illness, such as cardiovascular, respiratory, hepatobiliary, renal, hematologic, gastrointestinal, endocrine, immune, dermatologic, psychiatric, and central nervous system. Additional exclusion criteria included medical history and conditions that may affect the absorption, distribution, metabolism or excretion. Additionally, subjects with clinically significant abnormal values on clinical laboratory tests (blood hematology, urinalysis, and serum chemistry), 12-lead electrocardiograms, and a history of participation in any other clinical study within 60 days of the first administration of study drug were also excluded.

**Figure 1:**
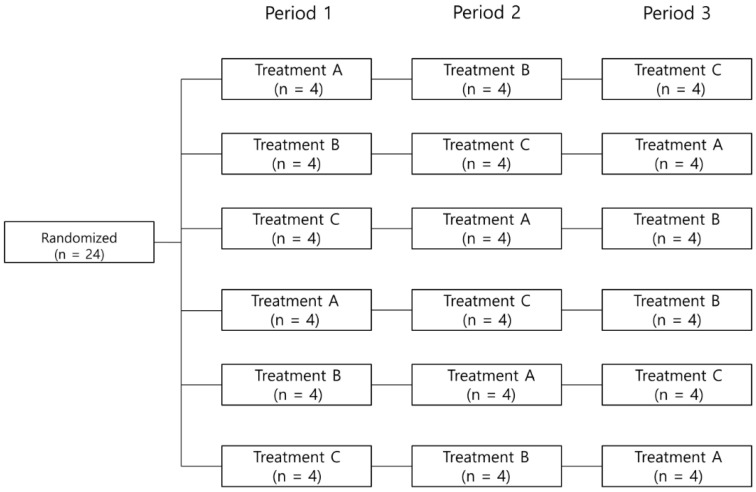
Study design. Subjects (*n* = 24) were randomized to one of the six sequence groups (four in each sequence in period 1). Subjects were administered the study drugs for 5 days with a 10-day washout between treatments.

This study was a randomized, open—label, multiple—dose, three—period, six-sequence, crossover phase 1 study conducted in healthy volunteers under fed conditions. Subjects received one of the three different treatments once daily for 5 days in each period: 160 mg micronized fenofibrate capsule (Lipilfen^®^, Daewoong Pharm. Co. Ltd., Seoul, Korea) alone (Treatment A), 2 mg pitavastatin (Livalo^®^, JW Pharmaceuticals, Seoul, Korea) alone (Treatment B), and co-administration of 160 mg micronized fenofibrate and 2 mg pitavastatin (Treatment C), respectively. There was a washout period of 10 days between treatments. Twenty-four subjects were randomly assigned to 1 of 6 treatment sequences, ABC, BCA, CAB, ACB, BAC, and CBA, as described in Figure 1.

All subjects were administered the study drugs with 240 mL of water every morning for 5 days to achieve the steady state. The drugs were administered at 30 min of completing the standardized breakfast (approximately 480 calories, 13% fat). In each period, all subjects came to the KNUH CTC every morning for 4 days for the study drug administration under the supervision of the investigator, following the assessment of vital signs. Subjects were admitted to the study center on the fourth day, 12 h before the last dosing in each treatment period, and were confined for 24 h after dosing. Additional visit after the last dosing by 48 h was made for pharmacokinetic sampling. 

Blood samples (9 mL each) were obtained at the scheduled time points of 0 (predose), 0.25, 0.5, 0.75, 1, 1.5, 2, 2.5, 3, 4, 5, 6, 8, 10, 12, 24, and 48 h after dosing on day 5. The samples were analyzed to measure the plasma concentrations of fenofibric acid and pitavastatin. In addition, predose blood samples were obtained on days 1 and 4 of each period. Blood samples were collected into tubes containing sodium heparin, and centrifuged (3000 rpm) for 15 min at 4 °C. The plasma samples were placed in Eppendorf tubes (1 mL each), a nd frozen at −70 °C or lower until they were analyzed by the analytical laboratory, Biocore Co. Ltd. (Seoul, Korea).

### 2.2. Bioanalytic Methods

Plasma samples for pharmacokinetic (PK) analysis were analyzed to determine plasma concentrations of fenofibric acid and pitavastatin by high performance liquid chromatography—tandem mass spectrometry (HPLC-MS/MS), and ultra-fast liquid chromatography (UFLC)-MS/MS, respectively, with some modifications of a validated method described in the literature [20,21,22].

In the fenofibric acid assay, chromatographic analysis (2795 Alliance HT HPLC system; Waters Corporation, Milford, MA, USA) was performed on an Atlantis dC18 column (3.0 μm particle size, 2.1 mm i.d. × 50 mm). The mobile phase was composed of a 65:35:0.1 (*v/v/v*) mixture of acetonitrile, deionized water, and acetic acid, with a flow rate of 0.2 mL/min. Detection of fenofibric acid was conducted by a Waters Quattro Premier XE mass spectrometer (Waters Corporation, Milford, MA, USA) with multiple reaction monitoring (MRM) in negative-ion mode at mass-to-charge ratios (*m/z*) of 317.0 → 231.0 and 323.0 → 231.0 for fenofibric acid and fenofibric-d_6_ acid, the internal standard (IS), respectively. Frozen plasma was thawed at room temperature, and vortexed for 10 s. After the addition of 10 μL of IS (50 μg/mL) to 100 μL of plasma in a polypropylene tube, 1 mL of acetonitrile was added and mixed for 1 min, and then centrifuged at 13,000 rpm for 5 min. Then, each 20 μL of the supernatant was mixed with 1 mL of 50% acetonitrile, and 100 μL of deionized water was added to 50 μL of the supernatant and mixed. A 10 μL aliquot of this solution was then injected into the LC-MS/MS system for analysis. 

In the pitavastatin assay, chromatographic analysis (Shimadzu Prominence UFLCXR system, Kyoto, Japan) was performed on a Unison UK-C18 (3.0 μm particle size, 2.0 mm i.d. × 75 mm). The mobile phase was composed of a 50:50:0.1 (*v/v/v*) mixture of 10 mM ammonium acetate, acetonitrile and acetic acid, with a flow rate of 0.25 mL/min. Detection of pitavastatin was conducted by an API-5000 mass spectrometer (AB SCIEX, Foster City, CA, USA) with MRM in positive-ion mode at *m/z* of 422.0 → 290.0 and 427.0 → 295.0 for pitavastatin and pitavastatin-d5, the IS, respectively. Frozen plasma samples were allowed to thaw at room temperature. After transferring 100 μL aliquot of plasma into the polypropylene tube, 25 μL of 100 mM ammonium acetate (pH 4.0 with acetic acid) was added to each tube, followed by the addition and mixing of 20 μL of the IS (100 ng/mL). After the addition of 1 mL of methyl tert-butyl ether, the tube content was extracted for 20 min and then centrifuged at 13,000 rpm for 5 min. The organic layer was evaporated to dryness under a stream of nitrogen gas. The residue was reconstituted with 200 μL of a 60:40:0.1 (*v/v/v*) mixture of deionized water, acetonitrile, and formic acid, and centrifuged at 2500 rpm for 3 min. A 3 μL aliquot of this solution was then injected into the LC-MS/MS system for analysis.

The lower limit of quantification was 1 μg/mL for fenofibric acid and 0.5 ng/mL for pitavastatin, and the linear calibration curves ranged between 1 and 60 μg/mL for fenofibric acid (r ≥ 0.9964), and between 0.5 and 200 ng/mL for pitavastatin (r ≥ 0.9997). The overall intra-day accuracy ranged from 98.2–115.6% for fenofibric acid, and from 89.2–115.2% for pitavastatin. The overall inter-day accuracy ranged from 106.5–110.0% and 94.3–106.9% for fenofibric acid and pitavastatin, respectively. The intra-day precision (% coefficient of variation (CV)) ranged from 1.2–9.2% for fenofibric acid and from 0.5–6.1% for pitavastatin. The inter-day precision (% CV) ranged from 3.8–7.4% and from 2.0–6.7% for fenofibric acid and pitavastatin, respectively.

### 2.3. Pharmacokinetic Analysis

Using actual sampling times via non-compartmental models with WinNonlin Pro 5.3 (Pharsight Corporation, Mountain View, CA, U.S.A), the PK parameters for fenofibric acid and pitavastatin were calculated based on the individual plasma concentration-time data as follows: the maximum plasma concentration during a dosing interval (τ) at steady state (C_max,ss_); the time to reach C_max,ss_ (T_max,ss_); area under the plasma concentration-time curve during a dosing interval after repeated dosing at steady state (AUC_τ,ss_), elimination half-life (t_1/2_), and total body clearance (Cl/F).

### 2.4. Safety of Subjects

The safety of pitavastatin and micronized fenofibrate were evaluated throughout the study period by assessment of subjective symptoms, vital signs, physical examination findings, results from clinical laboratory tests, and from 12-lead electrocardiogram testing that occurred on or after the administration of the first dose. In all subjects who received at least one or more doses of pitavastatin and micronized fenofibrate, treatment emergent adverse events (TEAEs) were evaluated and documented. All laboratory tests were performed at an accredited laboratory facility (Department of Laboratory Medicine, KNUH, Daegu, Korea).

### 2.5. Statistical Analyses

All statistical analyses were carried out using SPSS for Windows software (ver. 18.0; SPSS Korea, Seoul, Korea), and the level of statistical significance was defined as *p*-value below 0.05. Pharmacokinetic parameters were compared between the two treatment groups (co-administration versus individual drug administration) using a paired *t*-tests, or the Wilcoxon signed rank test. The geometric mean ratios (GMRs) and 90% confidence intervals (CIs) of log-transformed AUC_τ,ss_ and C_max,ss_ of fenofibric acid and pitavastatin for the two treatment groups (co-administration/individual administration) were calculated to evaluate the effect of co-administration of micronized fenofibrate and pitavastatin on the steady-state pharmacokinetics of each drug alone, using a general linear mixed model.

## 3. Results

### 3.1. Subjects

A total of 24 healthy participants were enrolled in the study following initial screening and randomly assigned to one of six different treatment groups. The study was completed without any subjects dropping-out. The means (ranges) for subject age, height, and weight were 24.5 years (20–31 years), 174.6 cm (161.0–187.0 cm), and 72.3 kg (53.9–91.8 kg), respectively.

### 3.2. Pharmacokinetics

**Figure 2:**
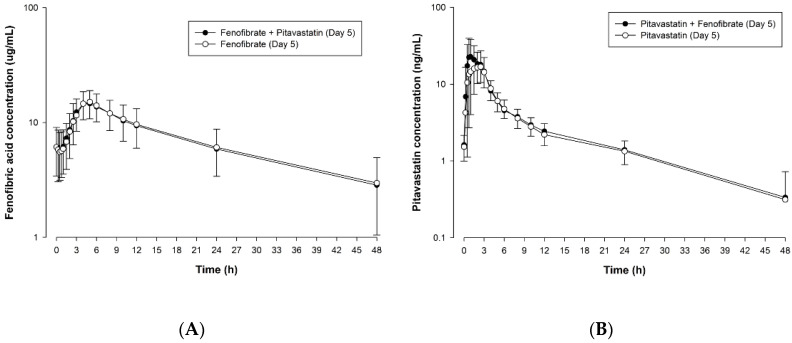
Mean (SD) plasma concentration-time profiles of fenofibric acid (**A**) and pitavastatin (**B**) after multiple oral administration of micronized fenofibrate or pitavastatin alone and co-administration of micronized fenofibrate and pitavastatin. Notes: “0 h” refers to 0 h on day 5 of each period.

The mean (SD) plasma concentration-time profiles for fenofibric acid and pitavastatin after 5 day repeated once daily co-administration of micronized fenofibrate and pitavastatin, or administration of each drug alone are illustrated in Figure 2A,B, respectively. PK variables for fenofibric acid and pitavastatin are summarized in Table 1. For fenofibric acid, the GMR value (90% CI) was 0.9827 (0.9295–1.0390) for C_max,ss_ and 0.9900 (0.9545–1.0269) for AUC_τ,ss_. For pitavastatin, the GMR value (90% CI) was 1.3576 (1.2056–1.5288) for C_max,ss_ and 1.1237 (1.0575–1.1941) for AUC_τ,ss_ (Table 2).

### 3.3. Safety

Multiple oral doses of 160 mg of micronized fenofibrate, and 2 mg of pitavastatin, administered concomitantly or individual administration, were well tolerated throughout the study. A total of 15 subjects (62.5% of 24 subjects) experienced at least one of the 40 reported TEAEs. Of all the 40 adverse events (AEs), 32 were determined to be possibly or probably related to the study medication (Table 3). Following the administration of the micronized fenofibrate alone, nine subjects (37.5% of 24 subjects) experienced at least one of the eight reported adverse drug reaction (ADRs) which included five cases of increased creatinine phosphokinase (CPK) levels, one case each of decreased white blood cells (WBCs), back pain, and both leg myalgia. In total, six subjects (25.0% of 24 subjects) experienced nine reported ADRs (four incidences of increased CPK, two incidences each of abnormal urinary occult blood and abnormal urinary RBC, and one case of diarrhea) following the administration of pitavastatin alone. After co-administration of micronized fenofibrate and pitavastatin, 10 subjects (41.7% of 24 subjects) experienced 15 reported ADRs, including 4 cases of elevated CPK, 3 cases of increased alanine transaminase (ALT), 2 cases of increased aspartate transaminase (AST) levels, and one case each of decreased WBC, hyperuricemia, increased LDH, abnormal urinary occult blood, abnormal urinary RBC, and diarrhea. All AEs were transient, and spontaneously resolved without specific treatment, with no severe or serious consequences. 

## 4. Discussion

This study evaluated the potential PK interactions, safety, and tolerability of combined treatment with micronized fenofibrate and pitavastatin compared with administering the drugs individually as multiple doses in healthy Korean male subjects. Peak and systemic exposure of fenofibric acid when co-administered with pitavastatin were comparable with those when micronized fenofibrate is administered alone. For pitavastatin, the AUC_τ,ss_ and C_max,ss_ values were increased by 12.4% and 35.8%, respectively, when it was administered in combination with micronized fenofibrate. 

According to the general dosing information, pitavastatin can be given at any time of the day with or without food, because the its absorption is not affected by food [6,8]. For the micronized fenofibrate, it should be administered with meals because its absorption is enhanced by approximately 35% under fed conditions when compared with administration under fasting conditions [12]. Accordingly, this study was conducted under fed conditions.

The maximum doses should be used in drug-drug interaction studies to maximize the possibility of identifying any possible interactions [23]. The maximum limit of the dosing regimens for micronized fenofibrate and pitavastatin are 160 mg and 4 mg, respectively [8,14]. The risk of myopathy may be enhanced with concomitant administration of fibrates with pitavastatin, and the combination therapy might lower the required dosage of each medication [8]. Accordingly, the doses selected in our study were 160 mg for the micronized fenofibrate, and 2 mg for pitavastatin, based on the assumption that maximum daily dose of 4 mg for pitavastatin would not be needed for the fixed-dose combination of micronized fenofibrate and pitavastatin.

In this study, the 90% CI values for the AUC_τ,ss_ and C_max,ss_ of fenofibric acid were 0.9545–1.0269 and 0.9295–1.0390, respectively, indicating that there was no significant changes in the fenofibric acid exposure when co-administered with pitavastatin. The 90% CI of the GMR for AUC_τ,ss_ (1.0575–1.1941) of pitavastatin fell within the range of 0.8000–1.2500, indicating that concomitant administration of fenofibric acid did not influence the extent of pitavastatin absorption at steady state. However, co-administration of pitavastatin with micronized fenofibrate resulted in 35.8% increase in the mean pitavastatin C_max,ss_, relative to pitavastatin alone. The increased C_max,ss_ of pitavastatin and earlier T_max,ss_ of pitavastatin in the presence of fenofibric acid in our study could be explained by the possible inhibition of intestinal P-gp-mediated transport of pitavastatin by fenofibrate [24].

The dose range of pitavastatin according to the prescribing information is 1 to 4 mg, and several studies has demonstrated the acceptable safety and tolerability profiles following the oral administration of 1–4 mg pitavastatin once daily [25,26,27]. Accordingly, the 1.36-fold increase in the mean pitavastatin C_max,ss_ after 2-mg pitavastatin co-administered with 160-mg micronized fenofibrate in this study is considered clinically not significant. 

Multiple doses of micronized fenofibrate and pitavastatin, whether co-administered together, or administered alone, were generally well tolerated in the healthy volunteers in this study. No subjects dropped-out because of the AEs, and there was no unexpected or serious AEs. According to the prescribing information of pitavastatin, concomitant administration of fibrates may increase the risk of myopathy [8]. However, the frequency of increased CPK, the most frequent AE, in this study, was similar whether the drugs were co-administered or administered separately as single agents. 

The study was conducted for a short period in a small number of healthy volunteers. Although the pharmacokinetic interactions and safety profiles of pitavastatin and fenofibric acid may differ from those in patients with dyslipidemia, several studies have shown tolerable safety profiles after pitavastatin and fenofibrate combination therapy. In a previous study conducted in patients with dyslipidemia receiving statin therapy, improved lipid profile and acceptable safety profiles has previously been reported after adding fenofibrate 200 mg for 8 weeks [28]. In another study for safety and efficacy of combination therapy of pitavastatin 1–2 mg daily for more than 2 months and micronized fenofibrate, 67 mg daily for 4–16 weeks, laboratory tests for liver, renal and muscle function were reported to be comparable to the effect of fenofibrate alone [29].

In conclusion, this study in healthy volunteers demonstrates that co-administration of 160 mg micronized fenofibrate and 2 mg pitavastatin had no clinically significant pharmacokinetic interactions, and there were no serious or unexpected AEs.

## Figures and Tables

**Table 1:** Steady-state pharmacokinetic parameters following administration of micronized fenofibrate (160 mg) and pitavastatin (2 mg) as concomitant administration versus individual administration under fasted conditions in healthy male subjects (*n* = 24).

**Variable**			***p*-Value ^‡^**
	**Fenofibric Acid**	**Fenofibric Acid + Pitavastatin**	
**Fenofibric acid**			
AUC_τ,ss_ (ng∙h/mL)	228.5 ± 76.3	225.1 ± 72.2	0.331 **
C_max,ss_ (ng/mL)	15.8 ± 3.7	15.5 ± 3.7	0.595 *
T_max,ss_ (h) ^†^	5.0 (2.5–6.0)	4.5 (2.0–6.0)	0.635 **
t_1/2_ (h)	20.7 ± 5.3	20.5 ± 5.1	0.689 **
CL_ss_/F (L/h)	0.8 ± 0.2	0.8 ± 0.2	0.837 *
	**Pitavastatin**	**Pitavastatin + Fenofibric acid**	
**Pitavastatin** AUC_τ,ss_ (ng∙h/mL)	106.1 ± 28.1	119.8 ± 34.4	0.007 *
C_max,ss_ (ng/mL)	22.4 ± 7.6	31.5 ±13.3	0.001 *
T_max,ss_ (h) ^†^	2.0 (0.5–4.0)	1.25 (0.33–3.0)	0.199 **
t_1/2_ (h)	15.2 ± 5.1	15.0 ± 4.2	0.817 *
CL_ss_/F (L/h)	20.6 ± 7.3	18.4 ± 6.5	0.002 **

Data are presented as mean ± SD except for T_max,ss_ values as median range ^†^. ^‡^ *p* value < 0.05, compared between the two groups by paired *t*-test * or Wilcoxon signed rank test **. Abbreviations: AUC_τ,ss_: area under the plasma concentration-time curve over the dosing interval at steady state; C_max,ss_: maximum plasma concentration at steady state; T_max,ss_: time to reach C_max,ss_; t_1/2_: terminal elimination half-life; CL_ss_/F, apparent clearance at steady state.

**Table 2:** Geometric mean ratio (90% CIs) for the log-transformed C_max,ss_ and AUC _τ,ss_ following administration of micronized fenofibrate (160 mg) and pitavastatin (2 mg) as concomitant administration versus individual administration in 24 healthy male subjects.

Variable	Geometric Mean Ratio (90% CI)	
	Fenofibric acid	Pitavastatin
AUC_τ,ss_	0.9900 (0.9545–1.0269)	1.1237 (1.0575–1.1941)
C_max,ss_	0.9827 (0.9295–1.0390)	1.3576 (1.2056–1.5288)

Abbreviations: AUC_τ,ss_: area under the plasma concentration-time curve over the dosing interval at steady state; C_max,ss_: maximum plasma concentration at steady state.

**Table 3:** Adverse drug reactions (ADRs) that were reported following multiple oral administration of 160 mg of micronized fenofibrate and/or 2 mg of pitavastatin in 24 healthy subjects.

System Organ Class/Preferred Term	Fenofibrate	Pitavastatin	Pitavastatin + Fenofibrate	Total
No. of events with ADRs	8	9	15	32
Investigations				
CPK increased	5	4	4	13
ALT increased			3	3
WBC decreased	1		1	2
AST increased			2	2
Hyperuricemia			1	1
LDH increased			1	1
Urinary OB abnormal		2	1	3
Urinary RBC abnormal		2	1	3
Gastrointestinal disorders				
Diarrhea		1	1	2
Musculoskeletal and connective tissue disorder				
Back pain	1			1
Both leg myalgia	1			1

Abbreviations: ADR: adverse drug reaction; CPK: creatinine phosphokinase; ALT: alanine aminotransferase; WBC: white blood cell; AST: aspartate aminotransferase; LDH: lactic dehydrogenase; OB: occult blood; RBC: red blood cell.
